# ADHD as Sequel to Acute Disseminated Encephalomyelitis

**DOI:** 10.15844/pedneurbriefs-30-2-6

**Published:** 2016-02

**Authors:** J. Gordon Millichap

**Affiliations:** 1 Division of Neurology, Ann & Robert H. Lurie Children's Hospital of Chicago, Chicago, IL; 2 Departments of Pediatrics and Neurology, Northwestern University Feinberg School of Medicine, Chicago, IL

**Keywords:** Attention-Deficit Hyperactivity Disorder, Acute Disseminated Encephalomyelitis, Behavioral Problems, Learning Disabilities

## Abstract

Investigators from Ruth Children's Hospital, Haifa, and Schneider Medical Center, Petah Tikuah, Israel, evaluated the long-term motor and neurocognitive outcome of 43 children hospitalized during 2002-2012 with acute disseminated encephalomyelitis (ADEM), and identified prognostic risk factors.

Investigators from Ruth Children's Hospital, Haifa, and Schneider Medical Center, Petah Tikuah, Israel, evaluated the long-term motor and neurocognitive outcome of 43 children hospitalized during 2002-2012 with acute disseminated encephalomyelitis (ADEM), and identified prognostic risk factors. Patients were treated with IV methylprednisolone 30 mg/kg for 3-5 days, followed by oral corticosteroids. Full neurological examinations and comprehensive neurocognitive and behavioral assessments identified different degrees of neurological sequelae in 26 children (61%) after a mean follow-up of 5.5 +/-3.5 years. Persisting symptoms occurred in 44%. The most common residual impairments included attention-deficit hyperactivity disorder (44%), behavioral problems (32%), and learning disabilities (21%). A full-scale IQ of 70 or less was found as a sequel to ADEM in five (12%) children, compared to 2.2% in the general population. Neurocognitive sequelae were found even in children considered fully recovered at time of discharge. The EEG performed in 16 patients was abnormal in 12 (75%); epilepsy developed in only 3 patients (7%). MRI revealed cortical and subcortical hemispheric lesions in 49% of patients, basal ganglia and thalamus in 31%, brain stem in 26%, spinal cord in 20%, and cerebellum in 15%. Complete resolution on follow-up MRI was found in 65% and partial resolution in 35%. Risk factors for severe neurological sequelae and poor long-term outcome were older age at presentation (p = 0.025) and male gender (p = 024). Neuropsychological testing and long-term follow-up is recommended for all children with ADEM, even in those with no neurological deficits at discharge. [1]

COMMENTARY. The cause of ADHD is highly genetic and involves the dopamine receptor and transporter genes. Environmental factors are also important and a gene-environmental interplay is frequently recognized [2]. Among acquired environmental etiologies for ADHD, viral infections linked to an increased risk of ADHD include measles, varicella, rubella, HIV, and enterovirus [2]. ADEM is uncommon. Apart from the above Israel study, only one similar study is listed in Pubmed. This involves 19 patients (10 with ADEM before the age of 5 years), reported from Australia. A brief neuropsychological assessment of children with ADEM compared to 19 controls showed that those with ADEM admitted before 5 years of age were particularly vulnerable to impairments in both cognitive and social domains, and had higher incidence of severe behavioral and emotional problems [3]. A further patient with severe deficits in arousal and sustained attention, associated with hemispatial neglect, were secondary to ADEM that involved the medial nuclei and pulvinar of the thalamus. Treatment with the noradrenergic agonist guanfacine was associated with a significant amelioration of impairments of both spatial and sustained attention [4]. Guanfacine XR is effective in treatment of ADHD [5], especially when stimulants are contraindicated.
